# Telemedicine and Virtual Health Care during Coronavirus Disease Pandemic

**DOI:** 10.31729/jnma.5204

**Published:** 2020-08-31

**Authors:** Badri Man Shrestha

**Affiliations:** 1 Sheffield Kidney Institute, Sheffield Teaching Hospitals NHS Trust, Sheffield, United Kingdom

The unprecedented Coronavirus disease 2019 (COVID-19) has overwhelmed the healthcare system globally and has caused serious disruption in its capacity to provide services to all categories of emergency, urgent and non-urgent patients. Since the COVID-19 outbreak is highly contagious, protection of patients and health workers from contracting COVID-19 and preservation of available resources such as hospital beds, staff and personal protection equipment (PPE) for treating COVID-affected patients are the main concerns to the healthcare delivery system. As recommended by the World Health Organisation (WHO), every nation has adopted strategies including lockdown, social distancing, isolation and home quarantine, hygienic measures, PPE and test and track protocol to mitigate the disease.^1^ To achieve these objectives, there have been introduction of telemedicine (TM) and virtual health care (VHC) as new models of delivering health care to avoid face-to-face consultation between patients and clinicians and thus reducing the risk of transmission of Coronavirus.

Telemedicine, a term coined in the 1970s, which literally means “healing at a distance”. The WHO has defined TM as “The delivery of health-care services, where distance is a critical factor, by all health-care professionals, using information and communications technologies for the exchange of valid information for diagnosis, treatment and prevention of disease and injuries, research and evaluation, and the continuing education of health-care workers, with the aim of advancing the health of individuals and communities.”^2^ Virtual health care is delivered without the patient and the clinician being present in the same physical location with utilisation of various digital communication modalities such as telephone and video. Combination of TM and VHC is termed as telehealth care (THC). The benefits of THC have been assessed in several countries. In the United Kingdom, USA and Australia adoption of THC was associated with a reduction in hospital admissions but, importantly, a significant improvement in participants' health literacy and health behaviours, together with improvements in anxiety, depression, and quality of life.^3^

In response to COVID-19 pandemic in China, patients were advised to seek physician's help online rather than in person and this proved significantly effective in reducing caseloads and in protecting patients and healthcare workers from contracting COVID-19. Following China's example, several countries including Italy, USA,UK, and India have embraced the THC technology and successfully reduced the need for “face-to-face” consultation and thereby the risk of transmission of Coronavirus. THC has been applied in all specialties of medicine effectively for monitoring, followup and treatment purposes. However, THC comes with a trade-off in the quality of patient care and learning opportunities to the doctors when seen in-person.^4^

Telemedicine has evolved during the pandemic and has been instrumental in reducing anxiety and panic within population and has allowed better allocation of resources through early triage of COVID-19 suspected patients based on their symptoms and test results. Patients with mild symptoms can be monitored at home through regular communication. Patients admitted in the hospital with severe symptoms are monitored from a different location with reduction of risk to the staffs. In Manchester, 350 patients with COVID-19 discharged from the hospitals were managed successfully at home through regular phone calls where patients were taught to record their oxygen saturation.^5^ To reduce risk of infection among health care professionals, hospitals have also implemented electronic intensive care unit monitoring.

To address the COVID-19 pandemic, several guidelines have been published in relation to adoption of THC, which highlight the criteria of patients suitable for THC, technology for use, data protection and the process of virtual consultation.^6,7^ THC can be used provided the patients are able and willing to communicate via telephone or video and that they do not need physical examinations or tests. Virtual consultation should not be used in patients with high-risk conditions requiring physical or internal (endoscopy) examination, unsuitable mental state (dementia) and with safeguarding concerns. Every hospital should adopt a uniform software platform for video consultations with appropriate hardware equipment and training for staffs. During the COVID-19 pandemic, the National Health Services of UK has procured a video platform known as “Attend Anywhere”, which is free of charge to all secondary care providers and is accessible to staffs who can remotely access, particularly from home in cases of self-isolation.^8^ It is convenient to use computers with virtual communications app (TEAMS) so that the patients can connect with a computer, or Android, or i-Phone device. Otherwise, telephone consultations provide a useful means of communication.

It is important to treat remote consultation as any other consultation in which sensitive and confidential information is safeguarded and all possible steps must be taken to reduce risk to patient confidentiality. The patients should be informed that the video and telephone consultations will not be digitally recorded without their prior consent, but the clinical outcomes will be recorded and stored on the patient records. If any intervention (medical or surgical) is already planned, it is mandatory to have discussion around consent with additional consideration in the context of COVID-19. Any delay in the delivery of service due to the pandemic and the balance of risk must be discussed. At the end of consultation, the encounter should be documented on patient's record including what information has been shared with the patient, what the patient has said and the agreed actions. A follow-up letter or email should be sent to the patient and appropriate health care team summarising the call and any action plan and timescales.

During the global pandemic with growing societal healthcare pressure, it is desirable to adopt telemedicine an alternative to traditional patient management system. The limitations for use, such as costs, training, and regulatory barriers, may limit the THC, but these can be circumvented in the current state of emergency through appropriate negotiations and education of the patients. The benefits of THC, including elimination of the risk of potential exposure of the patients and healthcare staff and maintenance of patient safety and expectations, makes a strong case for embracing the THC technology during the COVID-19 pandemic. This pandemic has already demonstrated the usefulness and reactivity of THC and constitutes an opportunity to embrace this technology into our health care systems in the long term.

## Conflict of Interest

**None.**

## References

[1] World Health Organisation. Coronaviris (COVID-19) advice to the public (2020). [Internet].

[2] World Health Organisation. A health telematics policy in support of WHO’s Health-For-All strategy for global health development (2020). [Internet].

[3] Fisk M, Livingstone A, Pit SW (2020). Telehealth in the Context of COVID-19: Changing Perspectives in Australia, the United Kingdom, and the United States. J Med Internet Res.

[4] Webster P (2020). Virtual health care in the era of COVID-19. Lancet.

[5] Thornton J (2020). The “virtual wards” supporting patients with covid-19 in the community. BMJ.

[6] General Medical Council - Remote consultation (2020). [Internet].

[7] Royal college of Surgeons of England - Virtual consultation (2020). [Internet].

[8] NHS England and NHS Improvement Implementing video consultations in NHS Trusts and Foundation Trusts: Frequently Asked Questions (2020). [Internet].

